# Cognitive impairment in heart failure is associated with altered Wnt signaling in the hippocampus

**DOI:** 10.18632/aging.102150

**Published:** 2019-08-25

**Authors:** Camilo Toledo, Claudia Lucero, David C. Andrade, Hugo S. Díaz, Karla G. Schwarz, Katherin V. Pereyra, Alexis Arce-Álvarez, Nicolás A. López, Milka Martinez, Nibaldo C. Inestrosa, Rodrigo Del Rio

**Affiliations:** 1 Laboratory of Cardiorespiratory Control, Department of Physiology, Pontificia Universidad Católica de Chile, Santiago, Chile; 2 Center for Aging and Regeneration (CARE-UC), Pontificia Universidad Católica de Chile, Santiago, Chile; 3 Centro de Investigación en Fisiología del Ejercicio, Universidad Mayor, Santiago, Chile; 4 Centro de Excelencia de Biomedicina en Magallanes (CEBIMA), Universidad de Magallanes, Punta Arenas, Chile

**Keywords:** aging, heart failure, cognitive impairment, Wnt signaling pathway

## Abstract

Age represents the highest risk factor for death due to cardiovascular disease. Heart failure (HF) is the most common cardiovascular disease in elder population and it is associated with cognitive impairment (CI), diminishing learning and memory process affecting life quality and mortality in these patients. In HF, CI has been associated with inadequate O_2_ supply to the brain; however, an important subset of HF patients displays CI with almost no alteration in cerebral blood flow. Importantly, nothing is known about the pathophysiological mechanisms underpinning CI in HF with no change in brain tissue perfusion. Here, we aimed to study memory performance and learning function in a rodent model of HF that shows no change in blood flow going to the brain. We found that HF rats presented learning impairments and memory loss. In addition, HF rats displayed a decreased level of Wnt/β-catenin signaling downstream elements in the hippocampus, one pathway implicated largely in aging diseases. Taken together, our results suggest that in HF rats CI is associated with dysfunction of the Wnt/β-catenin signaling pathway. The mechanisms involved in the alterations of Wnt/β-catenin signaling in HF and its contribution to the development/maintenance of CI deserves future investigations.

## Introduction

Chronic heart failure (HF) is a recognized health care problem affecting at least 26 million people worldwide [1]. HF is one of the most prevalent cardiovascular diseases in elderly and is a major cause of death, with a prevalence rising to ≥20% among people over 65 years of age [2]. Importantly, it has been documented a strong association between HF and cognitive impairments (CI) [3,4]. CI such as memory and global cognitive deficits affects 35-65% of patients with HF [5,6]. These cognitive alterations are likely to result in forgetfulness and poor learning ability, which may impair treatment adherence and sub-optimal self-care, increasing hospital admission and negatively affecting life quality and mortality in these patients [7,8].

HF can be classified according to the left ventricular ejection fraction (EF) in two subsets: HF with reduced EF (HFrEF), and HF with preserved EF (HFpEF) [9]. Notably, approximately half of the patients with HF have HFpEF. Furthermore, the relative prevalence of HFpEF appears to be increasing as the population ages [10,11]. Regardless of its etiology, HF patients and experimental models of HF exhibit enhanced sympathetic tone and sleep-related breathing disorders [12–15]. Interestingly, an increasing body of evidence suggest that the presence and severity of sleep breathing disorders and cardiovascular autonomic imbalance contribute to age-related CI, particularly in memory and learning process [16–18]. However, an import question related to HF and long-term cognitive decline is the role of EF in HF. Longitudinal studies showed that after HF diagnosis, rates of cognitive decline by EF category were not significantly different [19]. In HFrEF, CI has been associated with an inadequate cerebral perfusion (i.e. O_2_ supply to the brain), which may progress into neuronal cell death [20,21]. However, HFpEF patients also show CI [22]. Importantly, in HFpEF brain perfusion is preserved and cerebral oxygen supply is thought to be unaltered, then, the underlying mechanisms involved in CI development in HF must be distinct of mere oxygen delivery to the brain. Therefore, identifying the pathophysiological mechanisms that contribute to CI in HFpEF will help to develop future treatments intended to improve quality of life in these patients.

On the other hand, many studies indicate that CI in neurodegenerative diseases are closely associated with regional brain alterations, particularly in the hippocampus, a crucial structure in learning and memory processes [23]. Importantly, HF and age-related CI has been also associated with altered function of Wnt pathway (i.e. Wnt-responsive genes activation decrease) [24–26]. Particularly, Wnt pathway has been implicated largely in the regulation of synaptic assembly, as well as in neurotransmission and synaptic plasticity of hippocampal neurons [25,27,28]. In cardiovascular diseases, Wnt signaling has been proposed as a potential target for therapeutic intervention [29]. In HFrEF, Wnt signaling has been shown to promote adverse cardiac remodeling, cardiac hypertrophy and arrhythmogenesis [30,31]. Unfortunately, these results are often ambiguous when it comes to the question whether Wnt signaling in HFrEF should be activated or inhibited under pathophysiological conditions. Despite significant advances in the knowledge of Wnt signaling dysfunction in HF, there are no studies showing the alterations, if any, in the Wnt signaling pathway in the hippocampus in HF and its association with CI.

Taking account previous evidence, in the present work we aimed to assess the cognitive abilities and molecular mechanisms underpinning memory loss and learning decline, if any, in an experimental HF model without the confounding effect of chronic reductions in brain blood flow. Accordingly, we used a volume overload HF rat model that recapitulates the major pathophysiological hallmarks and diagnostic criteria for HFpEF [13]. We performed an integrative study evaluating CI in HF rats using behavioral and biochemical approaches. We showed for the first time that volume overload HF rats display memory loss and learning impairment compared with healthy age-matched rats. Additionally, we found that alterations in the Wnt/β-catenin pathway suggesting a possible role in the development/maintenance of cognitive deficits in HF rats.

## RESULTS

### Heart failure model

**Figure 1:**
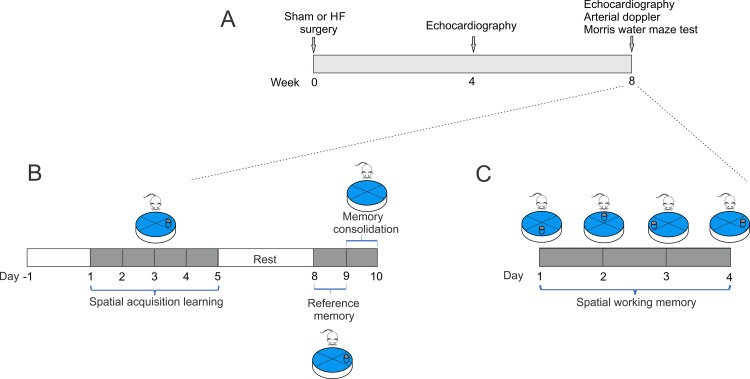
**Study timeline illustrating experimental design.** (**A**) At week 0, heart failure and Sham surgery were made. After 4 weeks, heart failure rats were evaluated by echocardiography to determine cardiac diameters. At 8-weeks, echocardiography, arterial doppler and procedures for assessing spatial and related forms of learning and memory were performed. (**B**) To determine spatial acquiring learning, rats were trained for two day, then tested for five consecutive days, followed by two days of rest, and three more days of testing. The 10th day, the platform was removed. (**C**) Followed spatial acquiring learning and after 2 days of rest, same set of rats performed memory flexibility test for four consecutive days and the position of the platform was changed daily.

**Figure 2:**
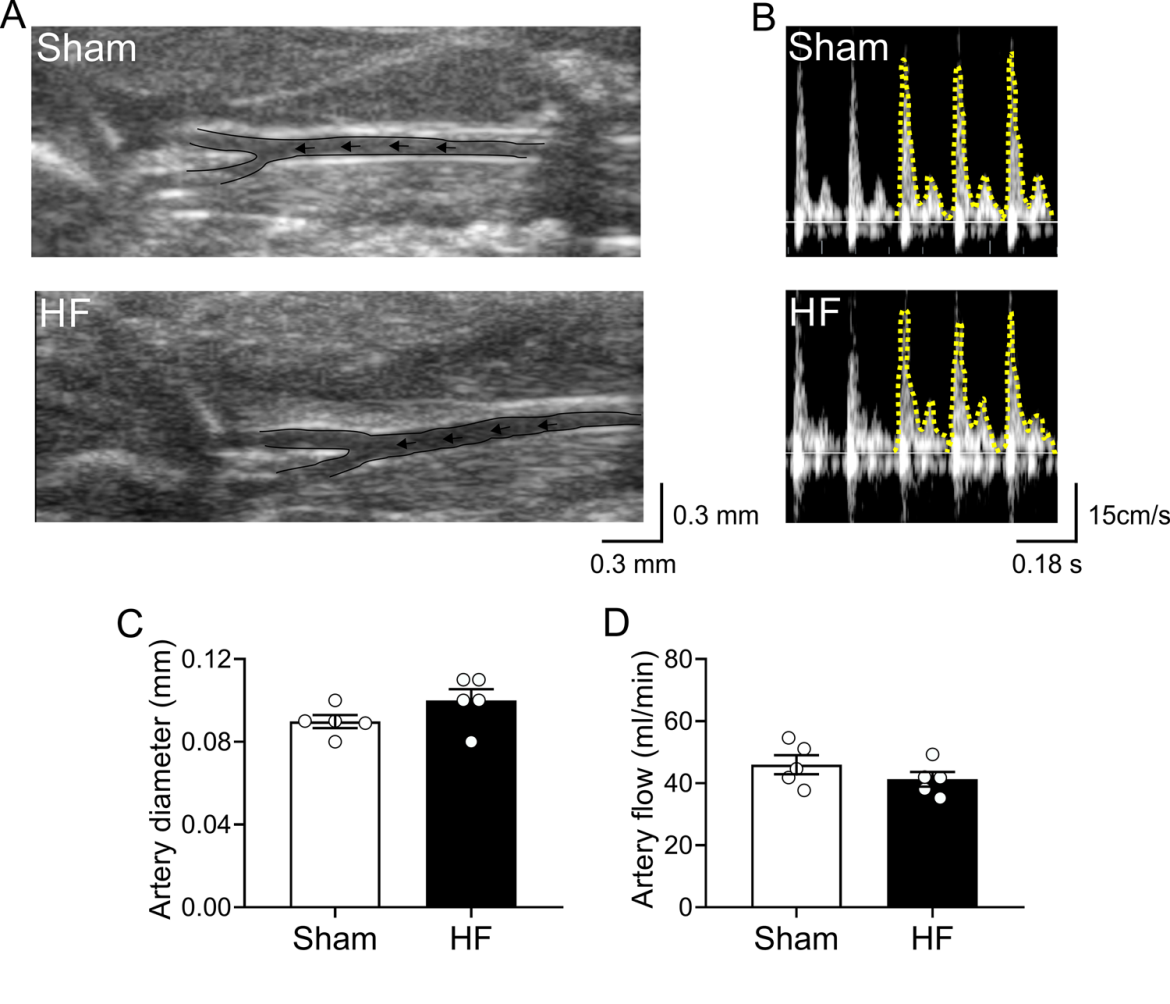
**Carotid artery blood flow in high output heart failure.** (**A**) Representative image of ultrasound scanning showing carotid artery in one Sham rat and one HF rat (**B**) Representative traces of carotid artery blood flow, assessed by Doppler at rest in one Sham rat and one HF rat. (**C**) Summary data showing carotid artery diameter. (**D**) Summary data showing carotid artery blood flow. Note that blood flow to the brain region was unaltered between groups. Values are means ± S.E.M. *p < 0.05 vs. Sham. Unpaired t-test. n=6 rats per group.

Experimental design and timeline of all assay are represented in Figure 1. Cardiovascular features of our HF model are shown in Table 1. Compared to Sham animals, HF rats displayed an increase end-diastolic diameter, end-diastolic volume, end-systolic diameter, end-systolic volume and stroke volume without significant differences in ejection fraction, (EF, 80.3 ± 5.3 vs 74.1 ± 8.9%; Sham vs. HF, respectively, p>0.05). Accordingly, HF rats showed overt signs of cardiac hypertrophy compared to Sham rats, as evidenced by a significant increase in the heart weight to body weight ratio (HW/BW, 2.7 ± 0.4 vs. 4.4 ± 0.5 mg*g^−1^; Sham vs. HF, respectively, p<0.05). No significant changes in body weight were observed between groups from the beginning to the end of behavioral tests (Table 1). In terms of cardiovascular baseline parameters, HF rats displayed a significant decrease in systolic blood pressure compared to Sham rats. Consequently, mean arterial blood pressure (MABP) was also decreased in HF rats compared to Sham rats (MABP: 101.1 ± 1.5 vs. 89.9 ± 8.3 mmHg; Sham vs. HF; p<0.05). In addition, HF rats displayed lower resting heart rate compared to control rats. In addition, carotid artery blood flow in Sham and HF rats was used as a surrogate for the estimation of blood perfusion to the brain region (Figure 2). No significant differences in both carotid artery diameter (Figure 2A and 2C) neither in carotid artery blood flow (Figure 2B and 2D) were found between Sham and HF group.

**Table 1:** Baseline cardiovascular parameters.

		**Sham** (n=6)	**HF** (n=6)				
BW (g) HW (g)		510.4 ± 12.8 1.4 ± 0.2	493.3 ± 15.5 2.2 ± 0.1*	
HW/BW (mg/g)		2.7 ± 0.4	4.4 ± 0.5*	
EDD (mm) ESD (mm) EDV (µl) ESV (µl) SV (µl) EF (%) SBP (mmHg) DBP (mmHg) MABP (mmHg) PP (mmHg) HR (beats/min)		6.0 ± 0.2 2.6 ± 0.1 180.0 ± 20.1 24.6 ± 4.0 155.4 ± 20.2 80.3 ± 5.3 118.3 ± 3.8 89.9 ± 6.2 101.1 ± 1.5 28.4 ± 2.5 309.1 ± 10.5	8.7 ± 0.2* 4.8 ± 0.2* 415.3 ± 25.6* 107.5 ± 8.2* 307.8 ± 19.2* 74.1 ± 8.9 101.5 ± 2.2* 79.1 ± 5.8 89.9 ± 8.3* 21.4 ± 2.1 280.6 ± 5.3*	
CO (ml/min)	48.0 ± 3.2			86.1 ± 5.4*				

Values are mean ± standard error of the mean (S.E.M.). BW: body weight; HW: Heart weight; EDD: end diastolic diameter; ESD: end systolic diameter; EDV: end diastolic volume; ESV: end systolic volume; SV: stroke volume; EF: ejection fraction: FS: fractional shortening; SBP: systolic blood pressure; DBP: diastolic blood pressure; MABP: media arterial blood pressure; PP: pulse pressure; HR: heart rate. T-test Student *, p<0.05 vs. Sham.

### HF rats display learning and memory impairment

**Figure 3:**
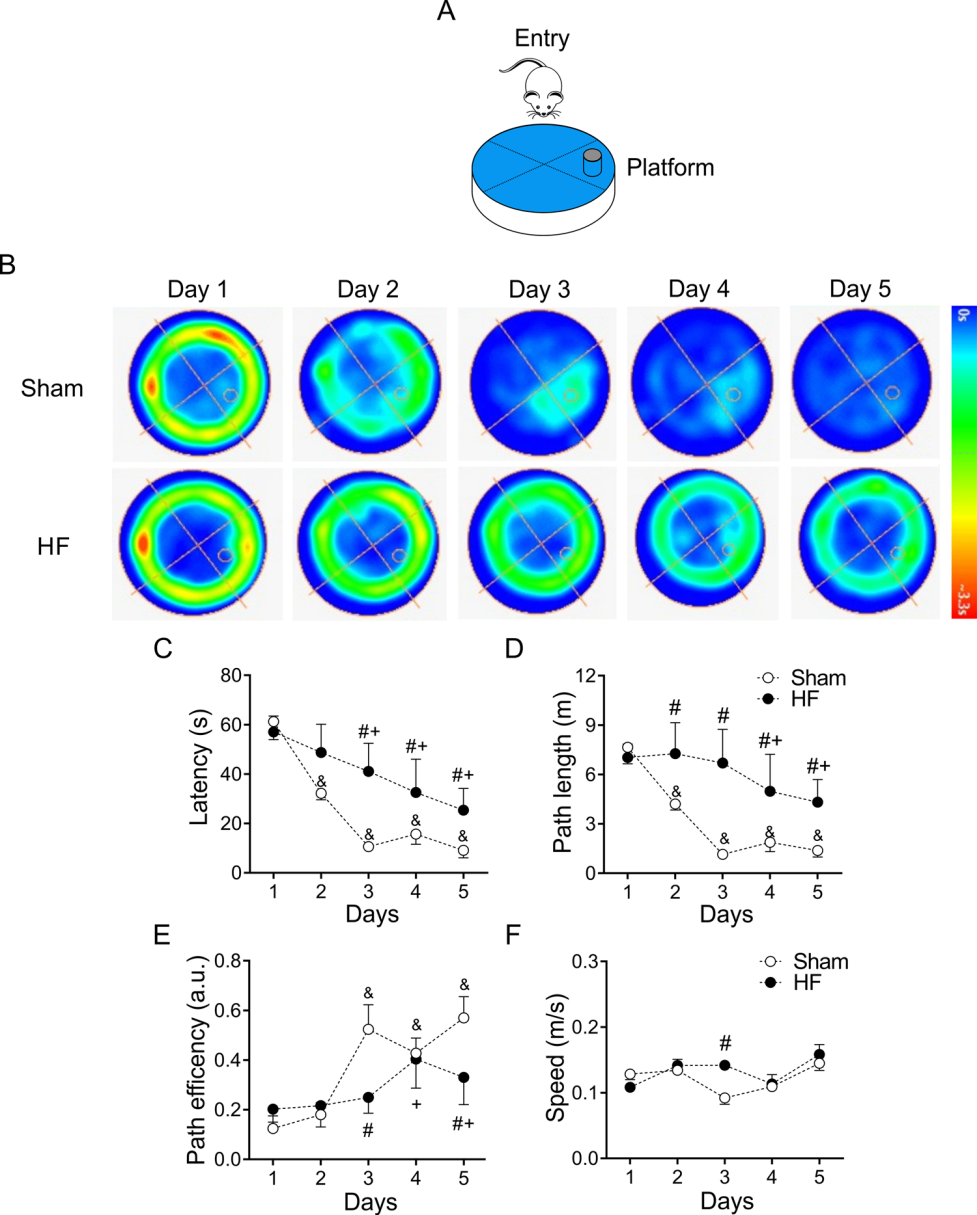
**Heart failure (HF) rats display spatial learning decline.** (**A**) Representative cartoon showing the rat and platform position in the pool. (**B**) Representative trajectory heat maps obtained from one Sham rat and one HF rat during Water Maze test between 1 to 5 days. Pseudocolor intensity indicate the time latency that the rat remained swimming in the pool searching for the platform. It is noteworthy that HF rats spends more time searching the platform across all experimental days compared to Sham animals. (**C**) Summary data of test duration during all experimental days. Note that HF rats required more time to find the platform. (**D**) Summary data of mean distance travelled during all experimental days. HF rats travel more distance compared to Sham animals. (**E**) Compared to Sham rats, HF animals display a decreased path efficiency in day 3 and 5. (**F**) Sham and HF rats display similar speed in all experimental days. &, p<0.05 vs. Sham day 1; +, p<0.05 vs. HF day 1; #, p<0.05 vs. Sham; two-way ANOVA following Holm Sidak test. n=6 rats per group.

The progressive decline of cognitive abilities is a defining characteristic of HF [20,32]. To evaluate changes in behavior, particularly in learning and memory function, we subjected healthy and HF animals to Morris water maze (MWM) test [33,34] (Figure 3A). Increased escape latencies to the target platform along the 5 trials days was observed in HF rats compared to control animals (100.0 ± 1.5 vs. 158.8 ± 1.9% of control; Sham vs. HF, respectively, p<0.05, Figure 3B). At first day of test, no changes in time, distance, or speed to target platform were found between groups, which ruled out the possibility that the spatial learning deficits detected in HF rats might be a product of deficient escape motivation or impairment of vision and/or motor skills [34]. During each following trial days, a significant increase in the time to reach the platform was observed in HF rats compared to Sham rats (Figure 3C). Consequently, the mean distance traveled to platform was increased in HF rats (Figure 3D). In addition, the actual path length divided by the direct path length, denominated path efficiency ratio, was higher in Sham rats compared to HF rats (Figure 3E). Swim speed along all trials days was not different between groups (Figure 3F). A significant decrease in mean times passing through the target platform and in the mean duration in the target quadrant were also observed in HF rats (data not shown). Interestingly, even significant changes in MWM measures were found between groups, the time elapsed and/or the distance travelled to reach the hidden platform showed a progressive decline in both HF and Sham rats (Figure 3).

**Figure 4:**
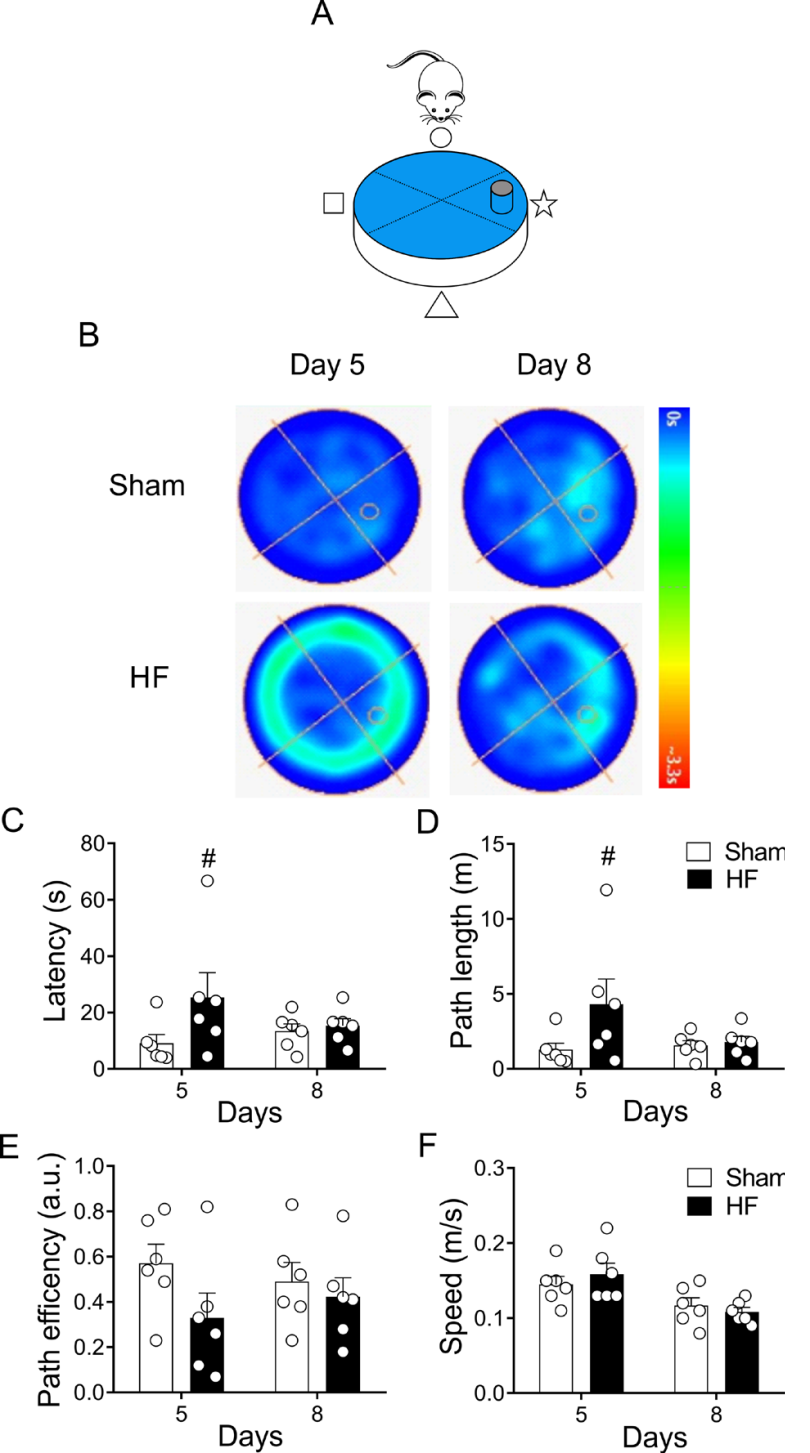
**Heart failure (HF) rats display similar reference spatial memory compared to control rats.** (**A**) Representative cartoon showing the rat and platform position in the pool. (**B**) Representative trajectory heat maps obtained from one Sham rat and one HF rat during Water Maze test between day 5 and day 8. Pseudocolor intensity indicates the time latency that the rat remained swimming in the pool searching for the platform. HF rats spends more time searching the platform in day 5; however, this behavior was absent in day 8. (**C**) Summary data showing that at day 5 HF rats need more time to find the platform; however, this effect was absent at day 8. (**D**) Summary data showing mean distance travelled during day 5 and day 8. At day 5 HF rats travel more distance compared to Sham animals. No significant differences in traveled distance was found between groups at day 8. (**E, F**) Sham and HF rats display similar path efficiency and speed in day 5 and day 8 of the test. #, p<0.05 vs. Sham day 5; two-way ANOVA following Holm Sidak test. n=6 rats per group.

On the other hand, place navigation requires the rats to learn to swim from starting position to the escape platform, thereby acquiring a long-term memory of the platform’s spatial location [35]. Accordingly, we assayed spatial learning 48 hours (day 8) after the last day of training sessions (day 5-8, Figure 1B). Following the trend observed in the heat maps trajectories (Figure 3), no significant changes in time, distance or path efficiency were found between groups at day 8 (Figure 4).

**Figure 5:**
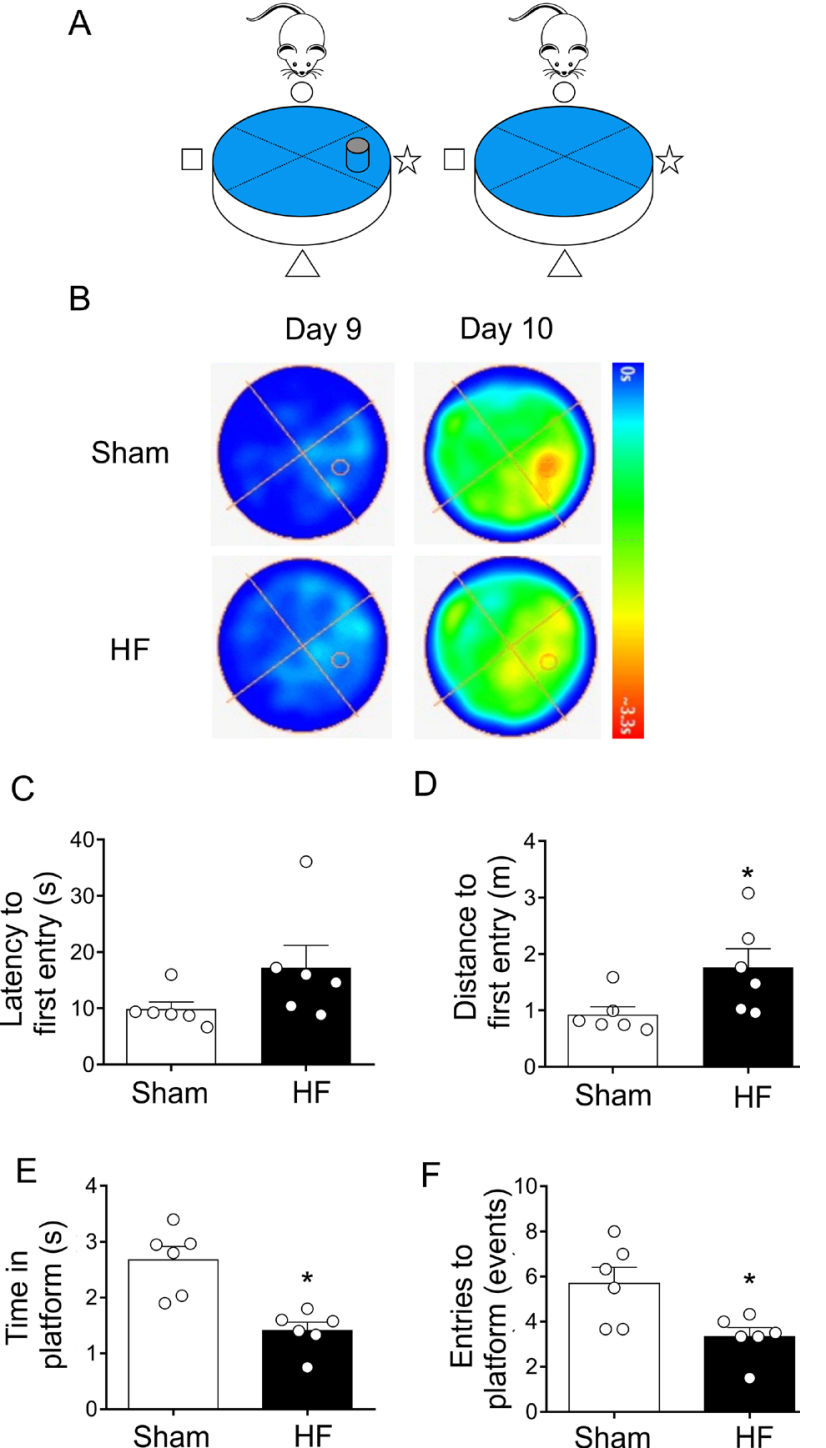
**Heart failure (HF) rats showed consolidation memory decline.** (**A**) Representative cartoon showing the rat and platform position in the pool. (**B**) Representative trajectory heat maps obtained from one Sham rat and one HF rat during Water Maze test during day 9 and 10. Contrary to day 9, in day 10 the platform was removed. Pseudocolor intensity indicate the time latency that the rat remained swimming and searching for the platform. Contrarily to Sham rats, HF animals showed less persistence in the place where the platform was originally located. (**C**) Summary data showing latency to first entry to the place where the platform was located (day 9). (**D**) Summary data showing the distance for first entry to the place where the platform was located (day 9). HF rats travelled more distance to first entry compared to Sham animals. (**E, F**) HF rats display a significant decrease in the time and entries to the place where the platform was located (compared to day 9). Values are means ± S.E.M. *, p<0.05, Unpaired t-test. n=6 rats per group.

The probe trial provides an index of the animal tendency to persist around the platform previous location and it is generally considered to be a measure of memory retention [33]. At day 9, both sham and HF rats learned where is the platform, therefore they reach the platform in less time. However, at day 10 the platform is removed from the pool to measure spatial bias (Figure 5A). Compared to Sham rats, HF rats displayed an increase in both, the latency to the first entry (Figure 5B and 5C) and in the swam distance to first entrance (Figure 5B and 5D) in the zone of the previous target quadrant. Additionally, the time spent in the target quadrant (or zone around the platform) (Figure 5A and 5E) and number of platform location crossings were both decreased in HF animals compared to Sham animals (Figure 5A and 5F). These data strongly suggest that HF rats display a loss of memory consolidation.

### HF rats display spatial memory deficits

**Figure 6:**
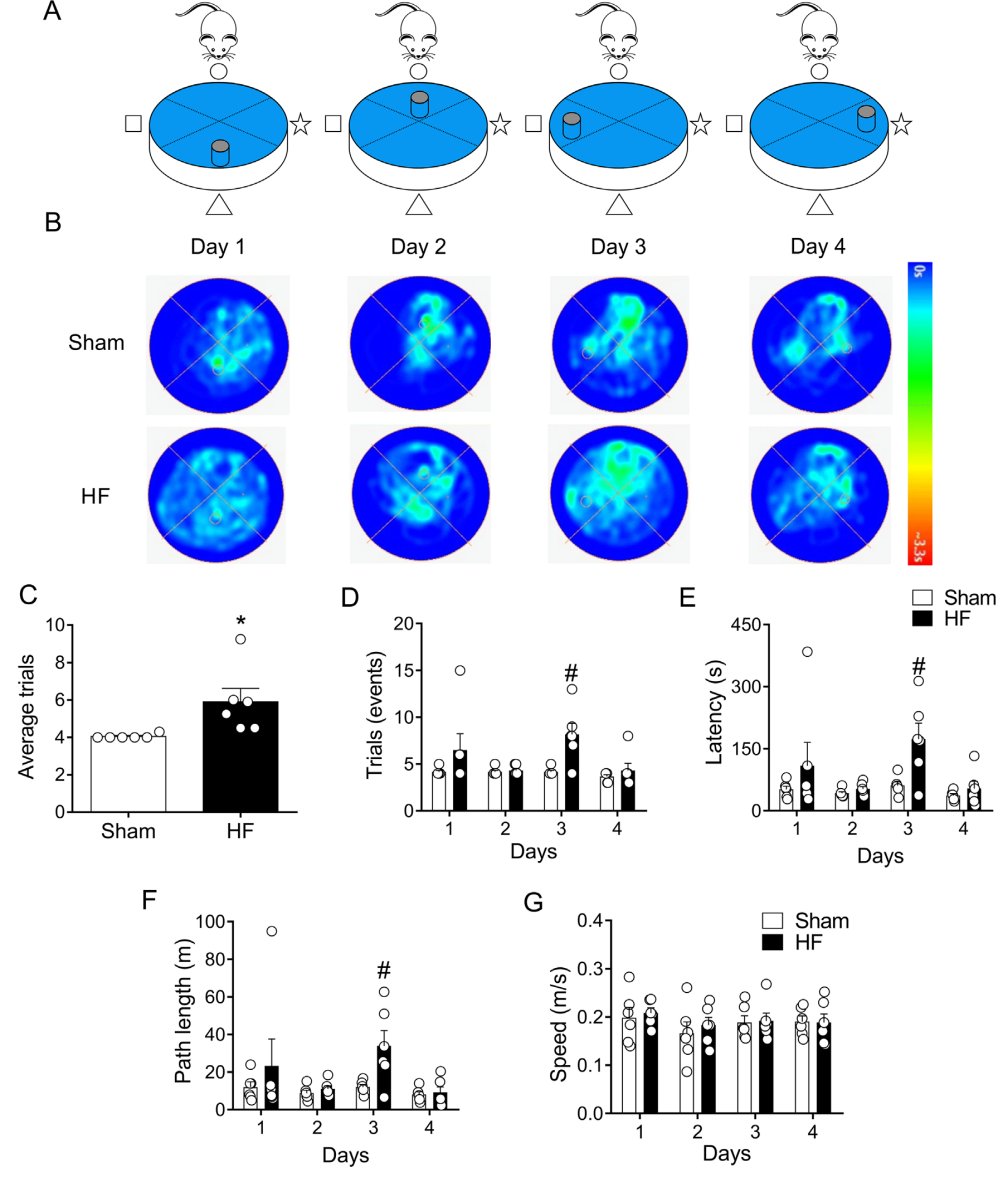
**Memory flexibility was impaired in heart failure (HF) rats.** (**A**) Representative cartoon showing the rat and different platform positions within the pool. (**B**) Representative trajectory heat maps obtained from one Sham rat and one HF rat during day 1 and day 4 of memory flexibility test. Note that during this test the platform position changed every day of the test. Pseudocolor intensity indicate the time latency the rat remained swimming in the pool searching for the platform. Contrarily to day 1, 2 and 4, at day 3 HF animals showed less latency in the platform position. (**C**) Average trials between day 1 and 4. Note that HF rats needed more trials to accomplish the task compared to Sham rats. (**D**) Summary data showing the number trials in each experimental day. (**E, F**) Summary data of the sum of latencies and paths length from each trial by day. Note that at day 3, HF rats display an increase test duration and distance travelled compared to Sham animals. (**G**) Average speed was not different in all experimental days between Sham and HF rats. Values are means ± S.E.M. *, p<0.05, Unpaired t-test; #, p<0.05 vs. Sham; two-way ANOVA following Holm Sidak test. n=6 rats per group.

After two days of rest, same rats were subjected to memory flexibility (MF) test, a variant of MWM (Figure 6A). This test measures the ability of the rats to find a new platform location using the procedural knowledge and spatial cues already learned in the reference memory version of the task [34]. As shown in Figure 6, Sham rats continuously decreased the number of trials required to complete the task, whereas rats with HF showed a reduced improvement when performing the task. Indeed, at day 3 of testing HF rats needed almost twice the number of trials to accomplish the task when compared to Sham animals (Figure 6D). In addition, a significant increase in time (Figure 6E) and mean traveled distance (Figure 6F) to reach the platform in each trial was observed in HF rats compared to Sham rats. This difference indicates that HF rats displayed memory impairment. No differences on average speeds when performing the tests were found between groups. This strongly suggest that HF rats and Sham rats displayed similar motor activity. Then, the data obtained strongly reflect memory impairment in HF rats.

**Figure 7:**
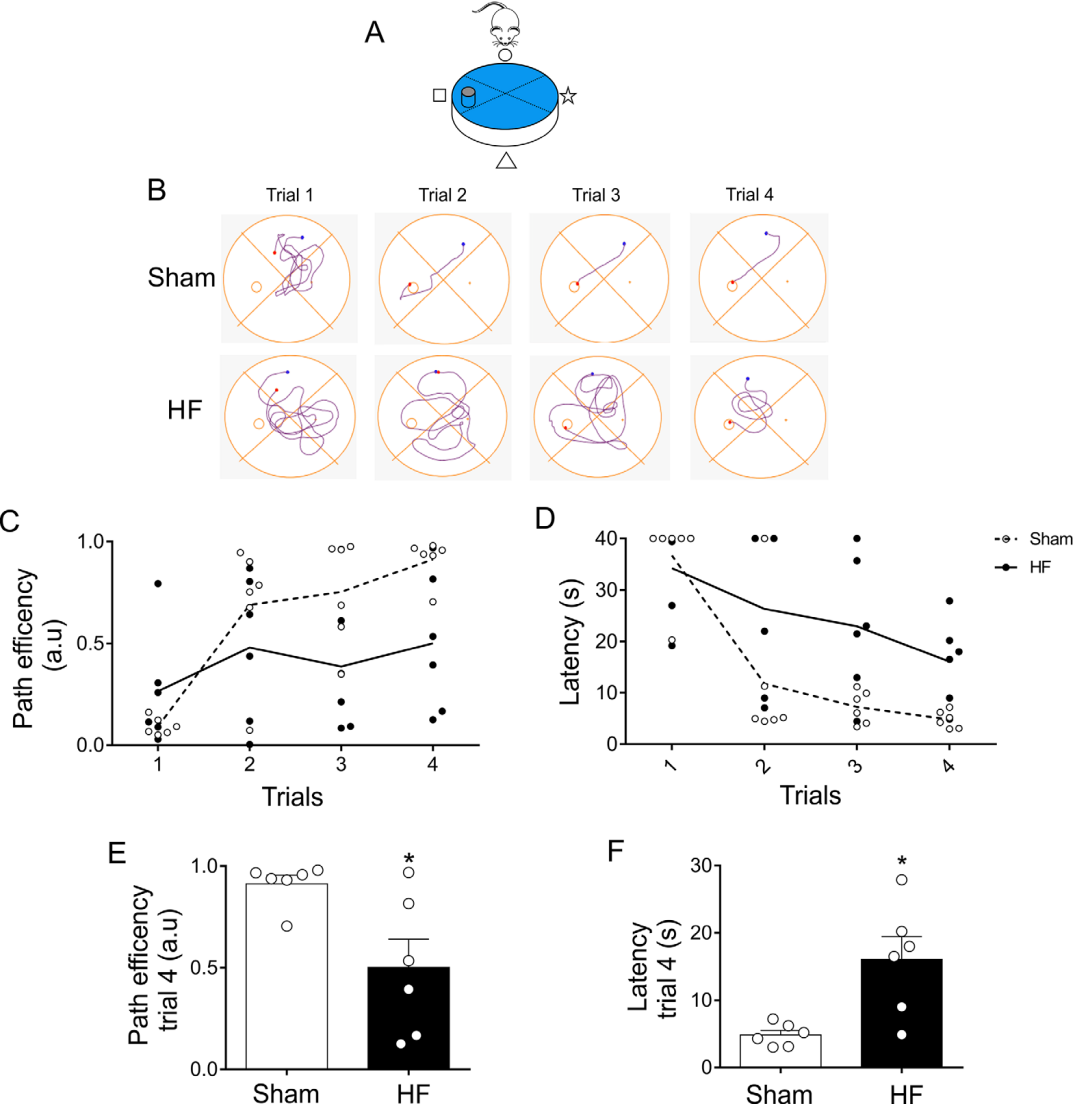
**Memory flexibility at day 3 was compromised in heart failure (HF) rats.** (**A**) Representative cartoon showing the rat and platform position at day 3. (**B**) Representative trajectory maps obtained from one Sham rat and one HF rat during day 3 of memory flexibility test. (**C**) Summary data showing average paths efficiency in each trial during day 3 of testing in Sham (white circle) and HF (black circle) rats. (**D**) Summary data showing average latencies from each trial during day 3 in Sham (white circle) and HF (black circle) rats. (**E, F**) Summary data of average paths efficiency and latencies at trial four (n=6 per group). Note that HF rats display an increase in the number of trials needed to complete the test, as well as an increase in the duration of each trial. Values are means ± S.E.M. *, p<0.05 vs. Sham, Unpaired t-test. n=6 rats per group.

At day 3 (Figure 1C), when experimental design allows to better characterize the search strategy the animal employs in memory trials (Figure 6A), HF rats displayed a significant decrease in mean path efficiency compared to Sham rats (Figure 7A-C). Accordingly, HF rats showed increases in the mean time required to reach the platform when compared to the times obtained in Sham rats (Figure 7D).

### Dysfunction of Wnt signaling in HF

**Figure 8:**
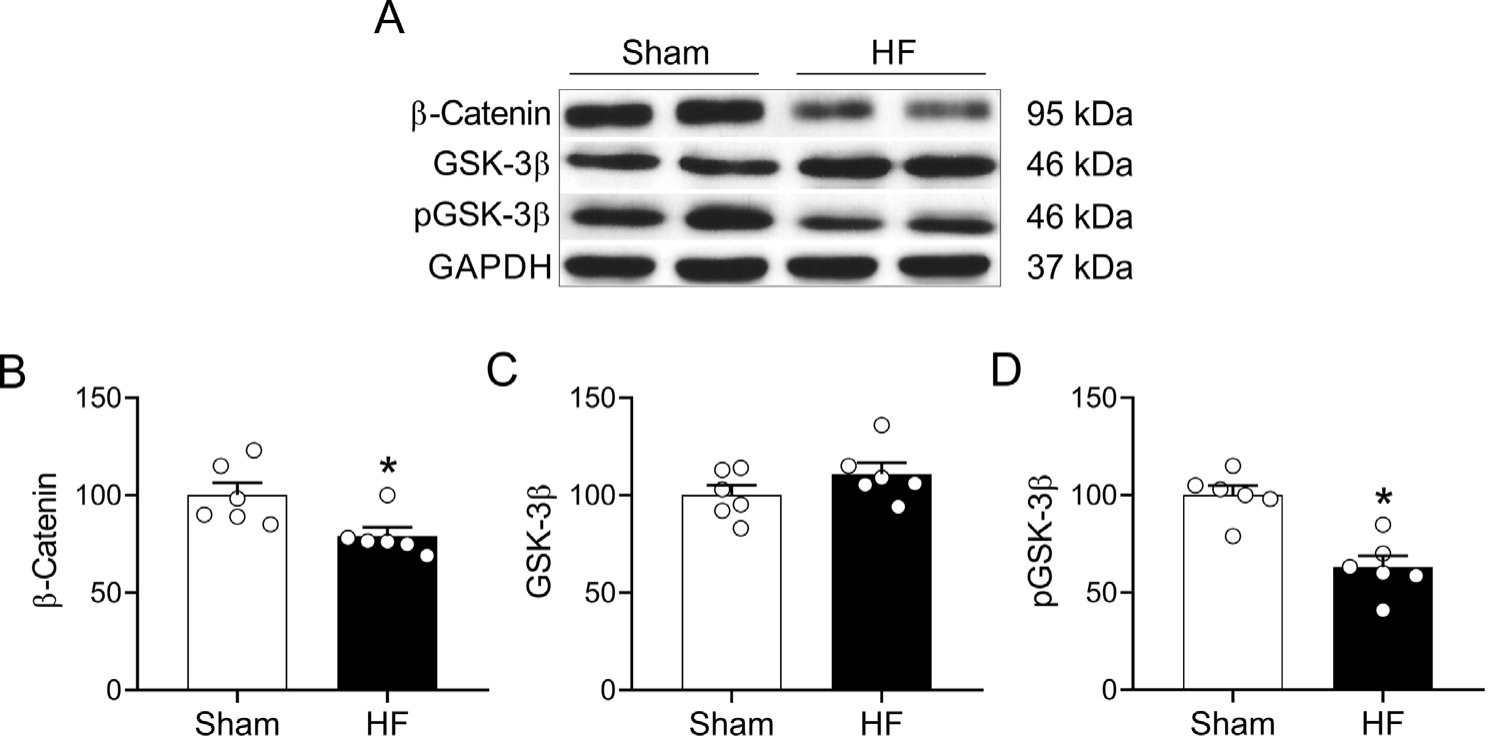
**HF rats display alterations in the Wnt signaling pathway in the hippocampus.** (**A**) Representative immunoblots showing the expression levels of active β-catenin, total GSK-3β and the inhibited form of GSK-3β (phospho-Ser9) in hippocampus micro-punches obtained from Sham rats and HF rats. (**B**) Summary data showing densitometric analysis of β-catenin, (**C**) total GSK-3β (D) and p-GSK-3β normalized against housekeeping protein GAPDH. Values are means ± S.E.M. *, p<0.05, Unpaired t-test. n=6 rats per group.

Wnt signaling play an important role in the development of the nervous system and participates in the adult brain structural and functional regulation of synapses [25,27]. Importantly, canonical Wnt signaling participate in hippocampal-dependent learning and memory processes [36]. Therefore, we evaluated whether HF may affect canonical Wnt signaling in the hippocampus. HF rats displayed decreased level of β-catenin (100.0 ± 6.3 vs. 79.1 ± 4.3% of sham; Sham vs. HF, respectively; p<0.05, Figure 8A and 8B) along with reduced level of phosphorylated GSK-3β (phospho-Ser-9) (100.0 ± 4.8 vs. 62.9 ± 5.9% of sham; Sham vs. HF, respectively; p<0.05, Figure 8A and 8D) suggesting an increased activity of the GSK-3β enzymatic complex. No changes in total GSK-3β levels were found between groups (Figure 8C).

## DISCUSSION

The major findings of this study are as follows: i) volume overload HF rats display altered learning process and memory loss, and ii) CI in HF rats was correlated to altered Wnt/β-catenin pathway evidence by a decrease in β-catenin levels and a reduction in the levels of phospho-Ser-9 GSK-3β. Together, our results strongly suggest that alterations in Wnt/β-catenin pathway in the hippocampus in HF rats are independent of blood perfusion to the brain.

### Volume overload HF model

The experimental HF model used in the present study is characterized by several features that closely mimic the pathophysiological consequences of HF in patients. Particularly, this model displays the characteristic neurohumoral activation of human HF, with increased sympathetic activity, breathing disorders and development of severe cardiac hypertrophy due to volume overload [37–41]. Importantly, we have previously shown that volume overload HF rats display increased end diastolic pressure volume relationship and end diastolic pressures without significant changes in ejection fraction, then no brain hypoperfusion is expected [15,42]. Also, important, all these hemodynamic features are considered diagnostic criteria for the recognition of human HFpEF [13]. Thus, in the present study we demonstrate for the first time the presence of CI in an experimental model of HF that recapitulates the major pathophysiological hallmarks and diagnostic criteria of HFpEF patients.

### Cognitive impairment in volume overload heart failure

Alterations in neuronal structure, loss of synapses, and dysfunction of neuronal networks are physiological processes associated with normal aging [23]. In HF patients, the occurrence of CI has often been viewed as coincidental events, because the prevalence of both HF and CI increases with age [4,10,43–47].

In the present study, we evaluate cognitive processes by conducting spatial learning and memory flexibility tests using the Morris water maze [48,49]. We found that HF rats presented a significative decrease in learning ability without overt evidence of sensorimotor deficits. These results suggest that decreased performance observed in HF rats is dependent on hippocampally-dependent mechanisms.

Interestingly, our data show that HF rats progressively learn; however, HF display slower learning rates compared to control rats. Accordingly, to assess reference retention at the end of the learning phase, a probe trial was performed 48 h after the last acquisition day (day 5-8). At current point we did not find statistical differences in latency and/or path length between groups, which indicate that HF rats reach a learning level similar to control rats. This may suggest then that HF rats and control rats shows no difference in reference memory (i.e. memory for the most recent training session) [34]. Finally, at day 10 (when the platform was absent) we found that HF rats display memory consolidation impairment as evidenced by a lower tendency to persist around the goal quadrant. In addition, working memory was also impaired in HF rats compared to healthy rats. Taken together, our data strongly suggest the presence of deficits in several hippocampal-dependent learning processes in HF rats.

On the other hand, the memory flexibility test showed that healthy animals constantly decreased the number of trials and time required to reach test criteria whereas HF animals showed little improvement in their performance (days 1 and 3). Presumably, at day 2, the fact that platform was located at the same quadrant where the animals were placed to begin the test made higher probability that the animals rapidly find the platform. At day 4, the platform was located at the quadrant it was placed during the spatial acquisition test (1 to 10 days); Then, it is plausible that animals first tended to swim directly to this position before beginning the exploration. Indeed, path efficiency at day 2 and 4 was very high for both groups. Importantly, day 3 is very relevant because it may better reflect short-term spatial memory since it was the only day in which the platform location was completely different compared to the tests performed previously in our experimental setting and it is not located close to the starting site (drop site) so no other confounding effects were expected. Because the average velocity was not different between groups, we can discard any motor activity failure in HF animals. As the platform is moved daily, no learning of platform position from the previous day can be transferred to the next day problem; hence, recall on each day during following trial is dependent on that day sample trial being the test a measure of temporary or working memory. Then, our data strongly suggest the presence of temporary/working memory deficits in HF rats.

### Wnt/β-catenin signaling in HF

HFpEF has been associated with an impaired cognitive function [10,22,50]. Brain perfusion has been linked to cognitive decline. Interestingly, neither brain perfusion impairment nor decreases in oxygen supply to the brain has been described in HFpEF patients. Therefore, it is possible that neurocognitive dysfunction in HFpEF is associated with alterations in neuronal networks and/or signalling pathways in cognitive-related areas within the brain independently of brain tissue perfusion. Wnt signaling pathway has been largely implicated in the regulation of synaptic assembly, neurotransmission and synaptic plasticity in the adult nervous system (for review see [27]). Indeed, impairment in synaptic plasticity by decreases in long term potentiation has been attributed partially to deregulation of Wnt signaling in hippocampal neurons [25,28]. Despite significant advances in the knowledge of Wnt signaling dysfunction in aging and neurodegenerative diseases, there are no studies showing alterations, if any, in the Wnt signaling during CI in HF. Importantly, our studies show for the first time that HF rats display a decrease in the active form of β-catenin levels and an increase in the activity of GSK-3β in the hippocampus, as indicated by the reduction in the levels of phospho-Ser-9 GSK-3β, its inactivated form. Importantly, these changes occur in HF rats which display learning impairment and memory deficits.

The underlying signal transduction mechanisms of HF-related disruption in Wnt signaling and CI are still unclear. However, we may speculate about the role played by adrenergic signaling on the alterations on Wnt signalling [24]. It is well known that β adrenergic receptors (β-Ars) activation increases cAMP and PKA. More importantly, PKA can regulate the activity of the canonical Wnt pathway [51,52]. Indeed, phosphorylation of β-catenin by PKA may enhance transcriptional activity by promoting β-catenin stability [53]. Furthermore, GSK-3β is inhibited through phosphorylation of serine 9 in GSK-3β by PKA [54]. Therefore, a loss in adrenergic pathway (i.e. β-ARs internalization) could play an important role in the control/regulation of Wnt signaling function being the outcome a decline in cognitive function. In experimental and human HF, increased discharges from the sympathetic nervous system results in increases in the systemic levels of catecholamines (i.e. norepinephrine, NE) [55,56]. Importantly, increases in NE spill over is the primary stimulus for β-ARs internalization in cardiac tissue. Therefore, it is plausible to hypothesized that during the progression of HF, increase levels of NE, which can also cross the blood brain barrier, may induced a loss of β-ARs signaling which may ultimately lead to the disruption of Wnt signaling through the regulation of β catenin and GSK-3β [24]. This interesting and novel mechanism deserves future investigations.

### Cognitive impairment in heart failure: does ejection fraction matter?

Most of the studies aiming to study cognitive decline in HF have been performed in HFrEF patients [5,57,58]. Particularly, studies have focused on individuals with cardiac systolic dysfunction and a left ventricular ejection fraction <40% since lower cardiac output in HFrEF may lead to inadequate cerebral perfusion, chronic hypoxic brain damage and/or neuroinflammation which all could account for structural or neurodegenerative changes which may progress into neuronal cell death [20,21,59]. Nevertheless, there is evidence showing that HFrEF patients do not display hypoperfusion to the brain; then, no hypoxic injury nor neuroinflammation exerted by low EFs is expected in HFrEF [22]. However, HFrEF patients do develop cognitive impairment [22]. Together, this evidence suggests that neurocognitive decline in HF is not completely related to brain perfusion [24]. In summary, decreased cerebral perfusion is recognized as a major mechanism related to CI establishment in HFrEF, however limited information is available about the molecular events responsible for CI observed in HFpEF, on which brain perfusion is preserved and cerebral oxygen supply is thought to be unaltered, despite an important subset of these patients show alterations in neurocognitive functioning [10,22,50].

Importantly, a recent longitudinal study showed that the rate of cognitive decline in patients with acute HF was independent on whether ejection fraction was reduced or preserved [19]. This evidence supports the notion of the existence of a common mechanism independent of EF, present in both HFrEF and HFpEF, that may trigger CI during disease progression. Interestingly, neurohumoral activation has been identified as a common factor between HF etiologies in both, human HFrEF and HFpEF as well as in experimental HFrEF and HFpEF [15,42,60–65]. Also, neurohumoral activation has been linked to neurovascular unit dysfunction [50]. Then, it is possible that neurohumoral activation in HF alters neuronal function and promotes cognitive impairment by means of neurovascular unit disruption in brain areas associated with learning and memory processes [24]. Future studies are needed to fully determine the role played by neurohumoral activation on CI in HF.

### Future perspective

CI in HF is associated with increased readmission rates in older patients and reduced quality of life [7,8]. Despite novel pilot trials have been performed in HFpEF patients to understand CI establishment in this subset of patients, their results are not conclusive, and further investigation is needed [19]. Then, understanding the mechanisms responsible for CI establishment in this condition could positively impact the current management of HFpEF suffering CI.

It is well documented that patients and experimental HF exhibit enhanced sympathetic tone and sleep-related breathing disorders, evidenced by alterations on specific regions of the brain involved in cardiorespiratory control [12,15]. Breathing disturbances (i.e. apneas/hypopneas) are considered a pathophysiological hallmark in HF, since they lead to arousals and sleep disruption events, which finally triggers an increase in sympathetic activity [66,67] ultimately adding more stress to the failing heart [68]. Interestingly, an increasing body of evidence suggest that the presence and severity of sleep breathing disorders contribute to age-related CI, particularly in memory and learning processes [16–18]. Indeed, breathing disturbances are frequent in Parkinson's disease (PD), Huntington's disease (HD) and Alzheimer's disease patients (AD) [69–71]. Moreover, it has been shown that patients with PD and AD exhibit marked abnormalities in cardiovascular autonomic regulation, evidenced by poor baroreflex gain [72] and parasympathetic/sympathetic dysfunction [73,74]. Then, disturbances in CNS cognitive areas (i.e. hippocampus) may occur in tandem or may be linked to alterations in cardiorespiratory control areas. Whether are cardiovascular/breathing disturbances triggering cognitive decline or vice-versa is an interesting topic that deserves further exploration.

### Additional considerations

Several limitations are inherent from our study. In patients with carotid artery stenosis, reduction of perfusion pressure due to HF with reduced ejection fraction (low cardiac output HF) may result in brain ischemic hypoperfusion lesions [75]. While brain hypoperfusion appears to be the major pathophysiological mechanism triggering cognitive impairment in low cardiac output HF [76,77], we cannot rule out the possibility that high cardiac output during volume overload HF may also contribute to the long-term cognitive decline by impaired vascular auto-regulation of the cerebral vasculature (i.e. hemorheological factors, increased resistance to flow, blood–brain barrier disruption). On the other hand, it has been shown that low systolic blood pressure is associated with cognitive impairment in patients with heart failure [43]. Consequently, lower blood pressure in cardiac diseases could contribute to decreases in blood flow to brain regions related to cognition [59,78,79]. However, cerebrovascular reactivity enables maintenance of normal perfusion even with severely elevated or decreased blood pressure and it protects the brain against blood pressure peaks. Therefore, the precise contribution of blood pressure, if any, on blood flow regulation and endothelial function in HF and its overall impact on cognitive function deserves future investigations. On the other hand, we focused on the hippocampus rather than in other brain regions (i.e. cerebral cortex, in the olfactory bulb and thalamus) because the most comprehensive evidence regarding the contribution of the Wnt/β-catenin signaling pathway on cellular and molecular aspects related to the development of cognitive impairment has been done in hippocampal neurons. However, this doesn’t mean that all the pathological processes are solely related to hippocampal alterations. Finally, although it is known that prevalence both neurodegenerative diseases such as AD, and cardiovascular diseases such as HFpEF have been increasing in females, we decided first to study cognitive decline in male high output HF rats, to avoid the confounding effect of hormonal status (for review see 80,81]). Therefore, further investigation in females will be required to determine the effect of sexual dimorphism.

## Conclusions

HF is the most common cardiovascular disease in the elder population and it is associated with neurocognitive impairment. In the setting of volume overload HF rat, a model of HF with preserved ejection fraction, we described decreases in learning process and memory loss, by mechanisms that seems to be independent of blood supply to the brain (HF model without changes in ejection fraction). In addition, we found that HF rats displayed a decrease in β-catenin levels and a decrease phosphorylation of GSK-3β suggesting an increase activity of this enzyme in the hippocampus. This study provides the first evidence showing alterations in hippocampus Wnt signaling in volume overload HF rats with overt neurocognitive decline.

## MATERIALS AND METHODS

### Animals

Adult male Sprague-Dawley rats (n=12), weighing initially ~250 g, were used in this study. All experiments were performed 8 weeks following heart failure induction. Rats were group housed (maximum 3 per cage) in a controlled temperature environment (22-25°C) with a 12-hour light/dark cycle and *ad libitum* access to food (Prolab RMH 3000, LabDiet, USA) and water, in accordance with the American Physiological Society, the National Institutes of Health guidelines for the Care and Use of Laboratory Animals and the Guía para el Cuidado y Uso de los Animales de Laboratorio from CONICYT. All experimental protocols were approved by the Ethics Committee for Animal Experiments of the Pontificia Universidad Católica de Chile and conformed to the principles and regulations as described by Grundy (2015) [82]. Efforts were made to limit the number of subjects and minimize animal suffering according to the principles of reduction, replacement and refinement in experimental design. At the end of the appropriate experiments, all animals were humanely euthanized by an overdose of anesthetic (sodium pentobarbital 100mg/kg i.p.).

### Rat model of HF

Volume overload HF was induced by the surgical creation of an arteriovenous fistula (A-V) using the needle technique as previously described) [15,37,41,42,65,83]. Briefly, under anesthesia (Isoflurane: 5% for induction; 2% for maintenance balanced with O_2_), the inferior vena cava and the abdominal aorta were exposed using a midline incision. Both vessels were clamped caudal to the renal artery and to the aortic bifurcation respectively. The aorta was punctured using an 18-gauge needle and advanced until it perforated the adjacent vena cava. Immediately afterward, a drop of histoacryl® glue (B. Braun, Germany) was used to seal the aorta at the puncture point. Sham-operated rats underwent the same anesthesia and surgical procedures but without performing the A-V fistula.

### Cardiac echocardiography and carotid artery blood flow doppler

Eight weeks after HF induction surgery, left ventricle cardiac diameters were evaluated using transthoracic echocardiography under light isoflurane anesthesia (5% for induction; 1.5% for maintenance balanced with O_2_). The criteria for HF was an increase in end-diastolic volume (EDV) and stroke volume (SV) (≥ 1.5-fold) relative to Sham without changes in ejection fraction (EF), as previously described [15,42,65,83]. Transthoracic M-mode echocardiography was recorded for quantification of cardiac dimensions at the level of the mid-papillary muscle with the parasternal short-axis view (Mindray Z6 Vet). The end-systolic diameter (ESD) and end-diastolic diameter (EDD) were averaged from 3 consecutive cardiac cycles according to the guidelines of the American Society of Echocardiography [15,42]. The end-systolic volume (ESV) and EDV were calculated using the Teicholz method ([ESV = 7*ESD^3^/(2.4+ESD)] and [EDV = 7*EDD^3^/(2.4+EDD)], respectively) [15,42,65,83,84]. The EF and fractional shortening (FS) were calculated from left ventricle volumes and diameters, respectively [15,42,65,76,77]. During the same session, rats underwent carotid artery Doppler to estimate blood flow [65].

### Hemodynamic measurements

Arterial blood pressure was measured with a conductance catheter in anaesthetized animals as previously described [15,42,85]. Briefly, A pressure conductance catheter (SPR-869; Millar, USA) was placed into the right carotid artery. After a 30 min equilibration period, baseline arterial blood pressure was recorded. Baseline hemodynamic parameters were: systolic blood pressure (SBP), diastolic blood pressure (DBP), pulse pressure (PP) and heart rate (HR). No significant differences in PaO_2_ has been previously showed between Sham and HF rats [15].

### Behavioral test

Eight weeks after HF induction surgery, all rats were trained in a circular pool for Morris Water Maze (MWM) test (1.6 m diameter, 75 cm deep and painted black) [34]. The room and water were maintained at 22°C. Spatial learning was assessed as previously described [26,34,86]. Briefly, rats were trained for one day, then tested for five consecutive days, followed by two days of rest, and three more days of testing (Figure 1). The tenth day, the platform was removed. Each rat performed three trials per day, with a maximum duration of 90 seconds or until the rat reached the platform. After each test, the rats were gently removed from the pool, dried and returned to their cage. All tests were capture using a video camera couple to Honestech TVR 2.5 program and the videos were analyzed with ANY-MAZE software. The parameters evaluated were: i) Latency: time from start to goal; ii) Path length: distance that the animal travelled during the test; iii) Average speed: average speed of the animal during the test, it was calculated by dividing the distance travelled by the test duration; iv) Path efficiency: Index of the efficiency of the path taken by the animal to get from the first position in the test to the last position. It was calculated as the straight-line distance between the first position in the test and the last position divided by the total distance travelled by the animal during the test. A value of 1 indicates perfect efficiency, values lesser than 1 indicate decreasing efficiency; v) Entries to platform: counts the number of events that the animal entered the platform; vi) Distance for first entry: distance travelled until first entry to platform; vii) Latency to first entry: length of time elapsed before the animal entered the platform for the first time; viii) Time in the platform: total amount of time the animal spent in the platform. Memory flexibility (MF) was assessed as previously described [34,49,87,88] (Figure 1). Briefly, followed spatial acquiring learning and after 2 day of rest the same set of rats performed the test for four consecutive days with a maximum of 15 trials per day and a maximum duration of 40 seconds per trial or until the rat reached the platform. The position of the platform was changed daily to a different quadrant during the experiment duration. Tests ended when the rat reached the platform in 20 seconds or less in three consecutive trials. After each test, the rats were gently removed from the pool, dried and returned to their cage. All tests were captured using a video camera couple to Honestech TVR 2.5 program and the videos were analyzed with ANY-MAZE software. The data is presented as: i) Trials: the number of trials after which the animals met the criteria; ii) Average trials: The average of all trials performed on all days of testing per rat; iii) Sum of latencies: Sum of time in which the test is performed, since the rat is placed in the pool until it reaches the platform in all trials per day; iv) Sum of paths length:Sum of distance that the animal travelled during each one trial per day; v) Path efficiency: Index of the efficiency of the path taken by the animal to get from the first position in the test to the last position per trial [34,35].

### Western blot

After euthanizing rats, brains were rapidly removed, and the hippocampus dissected on dry ice and stored at -80°C. Tissue was lysed in RIPA buffer containing 1% protease inhibitor cocktail (Sigma-Aldrich, USA) plus 1% phosphatases inhibitors (Phosphostop® Sigma, USA). Protein concentration was determined using Pierce™ BCA protein assay kit (Thermo Scientific, USA). Fifty micrograms of protein were separated by SDS-PAGE under reducing conditions on 10% acrylamide-bisacrylamide gels. Then, the proteins were transferred to a PVDF membrane (Millipore, USA), using the wet trans-blot system (Biorad, USA). Membranes were incubated in blocking solution, 5% non-fat milk diluted in Tris-saline buffer (TBS: 20Mm Tris base, pH7.2, 0.3M NaCl), for 1 hour at room temperature in constant agitation. Subsequently, the membranes were incubated with primary antibodies, mouse monoclonal β-catenin (E-5, sc-7963; Santa Cruz, Inc), mouse monoclonal total GSK-3β (sc-9166; Santa Cruz Inc), rabbit polyclonal GSK-3β (p-Ser9) (9336S; Cell Signaling), at a dilution of 1:1000 in blocking solution at 4°C overnight. Afterward, the membranes were washed with TBS with 0.1% tween-20 and followed by incubation with secondary antibody conjugated with HRP anti-mouse (074-1506, KPL) or anti-rabbit (074-1506, KPL) at a dilution of 1:2000 in blocking solution plus 0.1% tween-20 at room temperature for 1 hour. Later, the membranes were incubated with restore western blot stripping buffer (21059, Thermofisher) according to manufacturer instructions. Then, the membranes were incubated in blocking solution (5% non-fat milk in TBS) for 1 hour at room temperature at constant agitation. Subsequently, the membranes were incubated at 4°C overnight with loading control antibodies diluted in the same blocking solution (mouse anti-GADPH, 1:2000, sc-32233, Santa Cruz). Membranes were then washed in cold buffer solution and incubated with HRP conjugated anti-mouse antibody (1:2000, 074-1806, KPL). Proteins were visualized using ECL chemiluminescent solution (Pierce, USA) and exposed to high sensitivity film (BioMax MR Film of Carestream, Kodak). Finally, the signal intensities were quantified with the software image studio lite version 5.2 (Li-Cor Bioscience, USA). The protein levels were calculated based on the signal intensities of both proteins.

### Statistical analysis

Data was using Two-way ANOVA followed by Holm-Sidak post-hoc analysis or Unpaired Student’s t test according to data structure. The level of significance was defined as p <0.05. Results were presented as mean ± standard error of the mean (SEM). All statistical analysis was performed with GraphPad Prism 7.0 software (USA).
